# Epigenetic Therapies for Inflammatory and Immune-Mediated Skin Diseases

**DOI:** 10.3390/biomedicines14020373

**Published:** 2026-02-05

**Authors:** Anna Makridou, Dimitrios Iason Elemes, Maria Elpida Liakou, Paschalis Theotokis, Sofia Gargani, Efstratios Vakirlis, Soultana Meditskou, Alexandros Onoufriadis, Maria Eleni Manthou, Iasonas Dermitzakis

**Affiliations:** 1 School of Biology, Aristotle University of Thessaloniki, 54124 Thessaloniki, Greece; amakridf@bio.auth.gr; 2 Department of Histology-Embryology, School of Medicine, Aristotle University of Thessaloniki, 54124 Thessaloniki, Greece; delemes@auth.gr (D.I.E.); mliakoub@auth.gr (M.E.L.); ptheotokis@auth.gr (P.T.); sgargani@bio.auth.gr (S.G.); sefthym@auth.gr (S.M.); mmanthou@auth.gr (M.E.M.); 3 First Department of Dermatology and Venereology, School of Medicine, Aristotle University of Thessaloniki, 54643 Thessaloniki, Greece; svakirlis@auth.gr; 4 Laboratory of Medical Biology and Genetics, School of Medicine, Aristotle University of Thessaloniki, 54124 Thessaloniki, Greece; onoufriadis@auth.gr

**Keywords:** epigenetics, therapy, DNA methylation, histone modification, non-coding RNAs, psoriasis, atopic dermatitis, autoimmune diseases

## Abstract

Inflammatory and immune-mediated skin diseases are increasingly recognized as disorders in which genetic susceptibility is shaped and sustained by environmentally responsive regulatory programs. Psoriasis, atopic dermatitis (AD), vitiligo, systemic sclerosis (SSc), lupus erythematosus (LE), and lichen planus (LP) are clinically distinct, yet they share chronic or relapsing inflammation, tissue remodeling, and limited durability of many current therapies. Because genetic variation alone cannot fully explain disease onset, flare dynamics, heterogeneity in severity, or lesion recurrence, epigenetic mechanisms have emerged as a plausible link between environmental exposures and stable disease phenotypes in skin. Epigenetic regulation, including DNA methylation, histone modifications, and non-coding RNA networks, controls cell-type-specific transcription without altering the DNA sequence and may contribute to persistent inflammatory states and disease memory despite clinical improvement. The current review synthesizes primary preclinical and translational evidence on epigenetic-targeted therapeutic strategies across these conditions, focusing on interventions that modulate DNA methylation, histone acetylation and deacetylation, histone methylation, chromatin-associated regulatory proteins, and RNA-based approaches. We compare the maturity of therapeutic development across diseases, noting that research and intervention studies are concentrated in psoriasis and AD, whereas evidence for vitiligo, SSc, LE, and LP remains more limited and often derived from systemic or non-cutaneous models. Finally, we outline key gaps that currently restrict clinical translation and discuss why bridging them is essential for determining whether epigenetic modulation can move beyond proof-of-concept toward durable and clinically actionable interventions in inflammatory skin disease.

## 1. Introduction

Inflammatory and autoimmune skin diseases are increasingly regarded as conditions in which genetic predisposition is reinforced and perpetuated by environmentally driven regulatory mechanisms [1,2,3]. These conditions, including psoriasis, atopic dermatitis (AD), vitiligo, systemic sclerosis (SSC), lupus erythematosus (LE), and lichen planus (LP), are clinically distinct conditions, yet each is characterized by chronic or relapsing inflammation, tissue remodeling, and incomplete durability of many existing therapies [4,5,6]. While genome-wide association studies have clarified important risk loci, genetic variation alone does not adequately explain disease onset, flare dynamics, heterogeneity in severity, or the frequent recurrence of lesions at previously affected sites [1,3,7]. These limitations have shifted attention towards epigenetic mechanisms as the molecular interface that integrates environmental signals into stable disease phenotypes in the skin. Accordingly, environmental and lifestyle factors have emerged as important modulators of disease expression and contributors to comorbidities and psychosocial burden [8,9,10,11]. Despite these advances, many patients fail to achieve sustained remission with current therapies, underscoring the need for novel, targeted interventions [12,13,14].

Epigenetic regulation, which is classically encompassing DNA methylation, histone modifications, and non-coding RNA (ncRNA) networks, controls cell-type-specific transcription [15,16,17,18,19,20]. In the skin, these regulatory events coordinate keratinocyte differentiation, barrier integrity, melanocyte survival, stress responses, fibroblast activation, extracellular matrix deposition, as well as immune-cell polarization and memory [1,21,22]. Epigenetic programs integrate environmental cues with genetic susceptibility, shaping disease-relevant cellular states and functional programs [23,24]. Their plastic nature makes epigenetic programs attractive therapeutic targets, including DNA methylation and histone deacetylase (HDAC) inhibitors, microRNA (miRNA)-based modulators, and microbiome-derived interventions [16,18,25,26]. Epigenetic programs are inherently dynamic and, crucially, druggable. They are remodeled by inflammatory cytokines, oxidative stress, microbial cues, and environmental pollutants, yet remain anchored by dedicated enzymatic and chromatin machinery (writers, erasers, readers) that can be selectively targeted with small molecules or RNA-based therapeutics [17,19,20].

A central translational challenge in dermatology remains treatment durability, reflecting both recurrence of lesions despite clinical resolution and variability in patient responses [6,27,28,29]. Emerging multi-omic studies indicate that clinically improved tissue can retain disease-residual chromatin and transcriptional states, consistent with an epigenetic “memory” that primes rapid reactivation [25,30]. Preclinical work further demonstrates that manipulating epigenetic regulators can suppress pathogenic immune circuit such as T helper 17 cells (Th17)/interleukin 17 (IL-17) in psoriasis or Th2-skewed inflammation and barrier repression in AD, or reverse profibrotic fibroblast states in SSc [16,18,31,32]. However, despite this compelling mechanistic basis, clinical translation of epigenetic therapies remains in its infancy, highlighting the ongoing need for tailored approaches [1,3,7]. Collectively, these observations support the rationale for this review, highlighting epigenetic-targeted interventions as a promising approach in the management of inflammatory and autoimmune skin diseases.

In the current review, we synthesize primary preclinical and translational evidence supporting epigenetic-targeted therapeutic strategies across major immune-mediated and autoimmune skin diseases. We focus on interventions directed at DNA methylation machinery, histone acetylation and deacetylation pathways, chromatin reader proteins, histone methylation regulators, and ncRNA-based approaches. Where available, we also discuss human data linking epigenetic marks to therapeutic response and disease persistence. By outlining current experimental and clinical gaps, this review aims to serve as a roadmap for future advances in epigenetic therapy.

## 2. Overview of Epigenetic Mechanisms and Interventions

Three distinct yet interconnected mechanisms primarily govern the epigenetic landscape: DNA methylation, histone modifications, and ncRNAs [33,34]. Together, these mechanisms regulate the accessibility of DNA to transcriptional machinery, determining the transcriptional activation or repression of specific genomic regions [34,35].

### 2.1. DNA Methylation

DNA methylation is the most extensively characterized epigenetic modification, playing a fundamental role in genomic stability and the silencing of repetitive elements [36,37]. This epigenetic modification involves the DNA methyltransferase (DNMT)-mediated covalent transfer of a methyl group (CH_3_) from S-adenosyl methionine to the C5 position of cytosine, resulting in the formation of 5-methylcytosine (5mC) [2,25,36,37]. This process occurs predominantly within cytosine-guanine (CpG) dinucleotides, which are frequently clustered in regions known as CpG islands. CpG islands are enriched at gene promoters and other transcriptionally active regions and typically span 1000 bp [38,39]. Interestingly, CpG islands are present in approximately 70–80% of the human genome and play a critical role in transcriptional regulation. These marks are established by the de novo methyltransferases DNMT3A and DNMT3B and maintained during replication by DNMT1 [37,40]. DNMT1 mainly acts to preserve existing DNA methylation patterns, copying them onto the newly synthesized DNA strand during S phase by recognizing hemimethylated sites [41,42,43]. DNMT3a and DNMT3b function as de novo methyltransferases, initiating methylation at previously unmethylated genomic loci [42,44]. Although typically linked to transcriptional repression and gene silencing, DNA methylation is reversible, as ten-eleven translocation (TET) enzymes oxidize 5mC to 5-hydroxymethylcytosine (5hmC), initiating active demethylation pathways [37,45,46].

Across the studies reviewed, 5-aza-2′-deoxycytidine (5-Aza-dC) emerges as a prototypical DNMT inhibitor [47]. It incorporates into replicating DNA and covalently traps DNMTs, preventing the addition of methyl groups to cytosine residues and promoting passive DNA demethylation [48,49]. By reversing aberrant promoter hypermethylation, 5-Aza-dC restores the expression of key anti-fibrotic factors, reactivates epigenetically silenced genes, and exerts prominent anti-fibrotic effects, demonstrating broad epigenetic activity across in vitro and in vivo models of systemic sclerosis [50].

### 2.2. Histone Modifications

The nucleosome, the fundamental repeating unit of chromatin, consists of DNA wrapped around an octamer of histone proteins (H2A, H2B, H3, and H4) [33,35]. Histone amino-terminal tails undergo a wide range of post-translational modifications (PTMs), such as acetylation, methylation, phosphorylation, ubiquitination, and sumoylation, that regulate chromatin by altering histone–DNA interactions or recruiting “reader” proteins [16,51,52,53,54]. These PTMs create permissive or repressive states that control transcription, immune responses, and T-cell differentiation, e.g., IL-6 downregulation via DNA methylation and histone deacetylation [1,2,16,33,55]. Histone acetylation is regulated by histone acetyltransferases (HATs), which add acetyl groups to lysines to promote euchromatin, and HDACs, which remove them to compact chromatin and repress transcription [56,57,58]. HATs neutralize lysine charges, loosening histone–DNA interactions and enhancing transcription factor binding [51,52,59,60]. In contrast, HDACs reverse these effects, limiting regulator access and repressing transcription [35,51,52,53,61,62].

Bromodomain and extra-terminal (BET) proteins act as “readers” by binding acetylated lysines, recruiting transcriptional machinery, and sustaining inflammatory and lineage-specific transcription, notably via bromodomain 4 (BRD4) at promoters and super-enhancers [63]. On the other hand, histone methylation involves the enzymatic addition of one to three methyl groups to lysine or arginine residues on H3 and H4 N-terminal tails, thereby modulating chromatin and transcription factor access [64,65]. Polycomb Repressive Complex 2 (PRC2), via enhancer of zeste homolog 2 (EZH2), mediates H3K27 trimethylation (H3K27me3), establishing repressive chromatin by restricting transcription factor access, promoting compaction, and cooperating with DNMTs for long-term epigenetic memory [66,67]. Lysine methyltransferases catalyze, and histone demethylases reverse, these modifications, making methylation dynamic and reversible [68,69]. Polycomb group proteins in PRC1 and PRC2 further mediate chromatin compaction independently of their catalytic activities and catalyze H2A ubiquitination and H3K27 methylation, while PRC2 specifically recruits and cooperates with DNMTs to reinforce repression through DNA methylation [66,67,70,71,72,73,74]. Functional outcomes depend on residue and state: H3K4, H3K36, and H3K79 promote activation, whereas H3K9me3, H3K27me3, and H4K20 confer repression [51,52,75,76]. Small-molecule inhibitors targeting methylation, such as EZH2 inhibitors, reduce H3K27me3 and reverse repressive chromatin [77].

Consistent with this mechanistic framework, interventional studies targeting the regulation and recognition of histone acetylation have mainly used HDAC and BET inhibitors [78,79,80]. HDAC-directed interventions include HDAC inhibitors, such as vorinostat, trichostatin A (TSA), entinostat, remetinostat, RGFP966, and tubastatin A (TubA) [1,80,81,82,83,84]. Pan-HDAC inhibitors broadly enhance H3 and H4 acetylation by preventing deacetylation, whereas selective inhibitors sustain elevated acetylation in an isoform-, context-, and substrate-specific manner, affecting chromatin and non-histone protein function [85,86,87,88]. BET inhibitors targeting BRD2, BRD3, and BRD4 disrupt recognition of acetylated residues by epigenetic readers, impairing transcriptional machinery recruitment [79,89]. Prototypical compounds such as JQ1, together with second-generation and orally available BET inhibitors, have demonstrated robust anti-inflammatory and anti-proliferative effects in preclinical psoriasiform models by suppressing Th17-associated transcriptional programs and keratinocyte hyperactivation [66,90,91]. Moreover, with respect to epigenetic mechanisms, deacetylase complexes (such as NuRD) have been shown to facilitate PRC2 activity and H3K27me3 deposition, highlighting the crucial interplay between histone acetylation and methylation. Collectively, these interventions reshape the histone modification landscapes, enhance chromatin accessibility, and reset transcriptional programs via targeted modulation of methyltransferases, demethylases, and chromatin regulators.

### 2.3. Non-Coding RNAs (ncRNAs)

ncRNAs represent a multifaceted layer of epigenetic regulation that functions primarily at the post-transcriptional level [33,92]. This category includes miRNAs, long non-coding RNAs (lncRNAs), and circular RNAs (circRNAs), which do not translate into proteins but exert a profound influence over gene expression [16,93]. Furthermore, ncRNAs participate in complex feedback loops by modulating the expression of enzymes responsible for DNA methylation and histone modifications, thereby integrating post-transcriptional control with chromatin remodeling [94,95].

miRNAs are small, single-stranded RNA molecules, typically 21–23 nucleotides long, that constitute a pivotal layer of post-transcriptional and epigenetic regulation [25,96,97]. The human genome encodes over 1000 miRNAs, collectively controlling approximately 30% of protein-coding genes, with individual miRNAs capable of modulating hundreds of proteins and multiple miRNAs, often converging on the same target [34,98]. Following transcription by RNA polymerase II and processing via Drosha/DGCR8 and Dicer, mature miRNAs bind to 3′ untranslated regions (UTRs) of target mRNAs, eliciting gene silencing through mRNA cleavage, deadenylation, or translational inhibition [1,97,98]. miRNAs form a reciprocal regulatory network with the epigenetic machinery, modulating DNMTs, HDACs, and histone methyltransferases while being shaped by DNA methylation and histone marks, thereby controlling cell fate, chromatin dynamics, immune responses, intercellular communication, and representing promising therapeutic targets [1,97,98]. Another class of ncRNAs is the lncRNAs, which are transcripts longer than 200 nucleotides [99]. They typically lack protein-coding potential but act as key epigenetic regulators by modulating chromatin structure and gene expression through interactions with DNA, RNA, and proteins. Many lncRNAs function as molecular scaffolds, recruiting chromatin-modifying complexes such as PRC2 and DNMTs to specific loci, thereby influencing histone modifications (e.g., H3K27me3) and DNA methylation [100,101].

## 3. Epigenetic Interventions in Inflammatory and Immune-Mediated Skin Diseases

This section provides an overview of epigenetic therapeutic interventions evaluated in preclinical and clinical settings for major inflammatory and immune-mediated skin diseases, including psoriasis, AD, vitiligo, SSc, LE, and LP. The discussion is restricted to strategies aimed at skin involvement, acknowledging that for several of these conditions, the available evidence derives predominantly from systemic or non-cutaneous models. Across diseases, studies targeting DNA methylation, histone-modifying enzymes, chromatin reader proteins, and ncRNAs are critically reviewed to assess therapeutic potential, translational progress, and current limitations.

### 3.1. Psoriasis

Psoriasis is a chronic immune-mediated inflammatory skin disease characterized by epidermal hyperplasia, increased epidermal thickness, and dysregulated crosstalk between keratinocytes and immune cells, driven predominantly by the IL-23/IL-17 axis [18]. Genetic predisposition plays a major role, with HLA-C*06:02 representing the most robustly associated risk allele, especially in early-onset cases [102]. Accumulating evidence indicates that epigenetic mechanisms substantially contribute to disease initiation, maintenance, and variability in treatment response [103]. At the DNA methylation level, psoriatic lesions exhibit hypomethylation of promoters regulating inflammatory mediators and keratinocyte differentiation, alongside altered methylation patterns in immune cells that favor Th17 polarization. Histone modifications further shape the psoriatic transcriptome, with increased activity of HATs and reduced HDAC function promoting sustained expression of pro-inflammatory cytokines [104,105]. Given the consistent involvement of epigenetic pathways across epidermal and immune compartments, it is important to examine whether these mechanisms can be therapeutically modulated. The following section, therefore, summarizes primary preclinical and translational studies exploring epigenetic-targeted strategies in psoriasis [2].

#### 3.1.1. DNA Methylation

**Figure 1:**
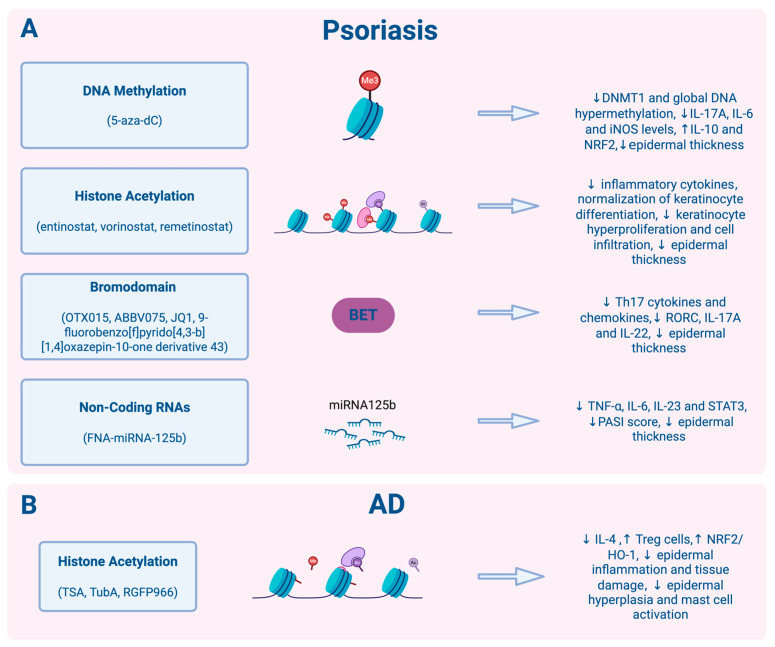
Epigenetic interventions in psoriasis and atopic dermatitis. (**A**) Psoriasis. Inhibition of DNA methylation using the DNMT inhibitor 5-aza-2′-deoxycytidine reduces epidermal thickness, leads to low IL-17A, IL-6, and inducible nitric oxide synthase (iNOS) levels, and normalizes aberrant DNA methylation patterns. Modulation of histone acetylation by HDAC inhibitors (entinostat, vorinostat, remetinostat) suppresses inflammatory cytokine production, reduces epidermal hyperplasia, and promotes normalization of keratinocyte differentiation. Inhibition of BET proteins (OTX015, ABBV075, JQ1, 9-fluorobenzo[f]pyrido [4,3-b][1,4]oxazepin-10-one derivative 43) attenuates Th17-driven inflammatory signaling, leading to reduced IL-17A, RORC and IL-22 and epidermal thickness. Non-coding RNAs further contribute to epigenetic regulation, with increased miR-125b levels associated with reduced inflammatory responses. (**B**) Atopic dermatitis. Modulation of histone acetylation with HDAC inhibitors (TSA, TubA, RGFP966) results in reduced epidermal inflammation and tissue damage. The downward arrow (↓) indicates downregulation (reduction) of the corresponding pathway, protein, or activity, while the upward arrow (↑) indicates upregulation of the corresponding pathway, protein, or activity. Created in BioRender. Dermitzakis, I. (2025) https://BioRender.com/lhfx2ce.

A preclinical in vivo study investigated whether pharmacologic inhibition of DNMTs could improve environmentally aggravated psoriasiform inflammation, a situation in which environmental exposures contribute to disease onset or progression [47]. In this study, the widely used imiquimod (IMQ)-induced psoriasis mouse model was exposed to the plasticizer di-(2-ethylhexyl) phthalate (DEHP) to generate an environmentally driven epigenetic model of psoriasis, in which DEHP-induced DNA methylation changes were associated with inflammatory responses. The IMQ mouse model is a widely used system, in which topical application of IMQ cream to a mouse induces symptoms that mimic human plaque psoriasis and is therefore commonly employed for drug screening. Mice were treated with the DNMT inhibitor 5-Aza-dC, which resulted in a marked reduction in epidermal thickness, inflammatory cell infiltration, and expression of key psoriatic cytokines (Figure 1). DNMT inhibition also reduced DEHP-induced epigenetic alterations by reducing global DNA hypermethylation in both dermal tissue and peripheral immune compartments, thereby restoring the expression of anti-inflammatory genes. Together, these findings establish a direct causal link between environmentally induced epigenetic dysregulation and psoriasis-like pathology in vivo. As this model reflects acute inflammation and short-term DNMT inhibition, questions remain regarding durability and safety.

#### 3.1.2. Histone Modifications and ncRNAs

BET bromodomain proteins act as epigenetic readers of acetylated histones, and their inhibition disrupts acetylation-dependent transcriptional programs [106]. An early in vivo study demonstrated that chromatin readers could serve as effective therapeutic targets in psoriasis. Authors evaluated the BET bromodomain inhibitor JQ1 in an IMQ-induced psoriasiform mouse model [90]. In this model, the BET inhibitor JQ1 significantly suppressed epidermal hyperplasia and Th17-associated cytokine production. Mechanistically, BET inhibition reduced transcription of the RAR-related orphan receptor C (RORC) and IL-17A, directly linking acetylated-histone “reader” proteins, which recognize epigenetic marks, to Th17 differentiation. Interestingly, elevated RORC expression has been consistently associated with enhanced Th17 responses and IL-17 pathway activation in psoriatic skin [107]. A human monocyte–keratinocyte co-culture system was also developed to screen the efficacy of BET inhibitors [108]. Multiple BET inhibitors significantly suppressed psoriatic inflammatory gene expression in vitro, and this suppression mirrored their therapeutic efficacy observed in vivo. In this context, the in vitro model demonstrated that BET inhibition directly modulates keratinocyte–immune cell cross-talk via histone acetylation-dependent transcriptional control. More recently, a novel orally available 9-fluorobenzo[f]pyrido [4,3-b][1,4]oxazepin-10-one derivative 43 displayed robust BRD4 inhibitory activity and pronounced therapeutic efficacy in an IMQ-induced psoriasis mouse model [91]. This agent improved ear thickness in a dose-dependent manner.

Given their central role in keratinocyte differentiation and immune activation, HDACs have emerged as attractive therapeutic targets in psoriasis. One preclinical study evaluated the pan-HDAC inhibitor vorinostat in primary human keratinocytes and in vivo in a chimeric mouse model of psoriasis, in which a psoriasis-like phenotype was induced in normal human skin grafted onto beige-SCID mice [109]. Treatment with vorinostat normalized keratinocyte differentiation markers and promoted apoptosis in primary keratinocytes. Using this chimeric mouse model, vorinostat was found to result in marked attenuation of a psoriasiform phenotype, with a significant decrease in epidermal thickness and inhibition of epidermal proliferation. Building on this work, topical HDAC1 inhibition was examined using entinostat in an IMQ-induced psoriasis mouse model [110]. The administration of this agent alleviated disease progression, accompanied by reduced infiltration of IL-17A^+^ γδ T cells and a marked decline in cutaneous psoriasis-associated cytokines. Within the psoriatic immune environment, entinostat not only restrained the IL-17A production by murine and human T cells and reduced IL-17-related transcripts in vitro but also interfered with the pathological dialogue between keratinocytes and T cells, leading to a substantial decrease in cytokines released by keratinocytes in co-culture systems.

More recently, remetinostat, a broader topical HDAC inhibitor, was examined in the same model [83]. This agent’s skin inflammation is associated with reduced CD86 levels on CD11C^+^ I-A/I-E^+^ dendritic cells within lesional tissue. In vitro, remetinostat also suppressed the maturation and activation of bone marrow-derived dendritic cells and partially reduced the production of psoriasis-associated inflammatory factors by keratinocytes. Beyond direct epigenetic intervention, histone modifications have also emerged as informative biomarkers of therapeutic response, linking existing psoriasis treatments to epigenetic regulation. Although these studies do not manipulate epigenetic enzymes directly, they demonstrate that effective biologic therapies induce—or are associated with—measurable epigenetic changes, reinforcing the clinical relevance of epigenetic pathways in disease control. Histone modifications were analyzed in peripheral blood mononuclear cells (PBMCs) from patients with psoriasis receiving anti-tumor necrosis factor (TNF) biologic therapy [111]. Distinct baseline H3K27ac and H3K4me3 profiles differentiated responders from non-responders at promoters of inflammatory genes. This human observational pharmacogenomic study assessed histone H3/H4 acetylation and H3K4/H3K27 methylation and identified differences between responders and non-responders in treatment-associated changes in histone methylation at early follow-up; it proposed these histone marks as predictive epigenetic biomarkers of biologic treatment response, while direct causal involvement has yet to be demonstrated. Another major layer of epigenetic intervention is non-coding RNAs, which regulate gene expression without altering the DNA sequence. One of the most recent approaches involves RNA-based epigenetic modulation. A topical delivery system for miR-125b was developed using framework nucleic acids and evaluated in an IMQ psoriasis mouse model [112]. Topical delivery of miR-125b via functional nucleic acid (FNA) significantly reduced epidermal hyperplasia and inflammatory cytokine expression in lesional skin. This nanotherapeutic study supports RNA-based epigenetic intervention but lacks long-term toxicity and stability assessment.

Collectively, primary studies demonstrate that DNMT inhibitors (e.g., 5-Aza-dC), HDAC inhibitors (e.g., entinostat, remetinostat, vorinostat), BET inhibitors (e.g., JQ1), and miRNA-based topical therapies (e.g., miR-125b) exert significant anti-psoriatic effects in preclinical models. However, the field remains at a very early stage, with interventional clinical trials of epigenetic drugs in humans being scarce and long-term safety largely unexplored, highlighting the need for robust translational studies to bridge preclinical insights to clinical application.

### 3.2. Atopic Dermatitis (AD)

AD is a chronic, relapsing inflammatory skin disease clinically characterized by intense pruritus, eczematous lesions, epidermal barrier dysfunction, and a strong association with allergic comorbidities [36]. Beyond its well-established immunologic hallmarks, namely Th2-inflammation and cytokines such as IL-4, IL-13, and IL-31. Recurrent scratching perpetuates an itch–scratch cycle that substantially impairs patients’ quality of life [113]. AD is increasingly recognized as a disease in which environmental exposures and genetic susceptibility converge through epigenetic regulation [114]. Histone modifications and ncRNAs act as molecular “integrators” of environmental triggers—such as allergens, pollutants, and microbial signals—into sustained inflammatory and barrier-defective states in AD skin [115]. Importantly, these epigenetic alterations are not merely descriptive; they correlate with clinical outcomes, including disease chronicity, flare frequency, and response to both topical and systemic therapies [7]. This has positioned epigenetic regulators not only as mechanistic drivers of AD pathophysiology, but also as attractive therapeutic targets. As outlined in the sections below, preclinical studies have begun to test epigenetic interventions directly, while parallel human studies suggest that existing therapies may exert part of their efficacy through epigenetic interventions [116,117].

Histone deacetylation plays a central role in amplifying inflammatory transcription in AD and has emerged as a key therapeutic target. Early preclinical evidence evaluated the pan-HDAC inhibitor TSA in a murine model, induced by 2,4-dinitrofluorobenzene (DNFB), which is widely used as it mimics inflammatory conditions similar to those present in the skin of patients with AD [118]. This work established HDAC as a defined epigenetic therapeutic target in AD, demonstrating that TSA treatment improved dermatitis and epidermal hyperplasia. More recently, HDAC3 inhibition was investigated in a 2,4-dinitrochlorobenzene (DNCB)-induced AD mouse model [119]. Pharmacologic HDAC3 selective inhibitor, namely RGFP966, rescued the suppressed nuclear factor erythroid 2-related factor 2 (Nrf2)/heme oxygenase-1 (HO-1) signaling activity in lesional skin and markedly reduced epidermal inflammation and inflammatory cell infiltration. This study linked histone deacetylation to oxidative-stress-driven AD pathology. Additionally, pharmacologic HDAC6 inhibition with TubA significantly improved clinical symptoms in a DNCB-induced AD model, prevented AD-associated epithelial hyperplasia, and reduced the number of activated mast cells [84].

Current evidence indicates that epigenetic dysregulation in AD primarily involves histone modifications, which amplify inflammatory transcription and mediate immune–epidermal crosstalk. Preclinical studies show that HDAC inhibitors, including TSA, RGFP966, and TubA, reduce inflammation, immune-cell recruitment, and oxidative stress in experimental AD models. In contrast, epigenetic strategies targeting DNA methylation or ncRNAs remain limited. All current interventions are still at the preclinical stage.

### 3.3. Vitiligo

Vitiligo is an acquired depigmenting disorder characterized by progressive melanocyte loss driven by oxidative stress, innate immune activation, and autoreactive cytotoxic T cells [120]. It usually appears as well-defined white patches on the skin, commonly affecting the face, hands, and other exposed areas [121]. The pathogenesis of non-segmental vitiligo is shaped by genetic polymorphisms affecting immune regulation and melanogenesis-related pathways [122]. In contrast to other inflammatory skin diseases, such as psoriasis and atopic dermatitis, there are currently no studies investigating defined pharmacological epigenetic interventions in patients with vitiligo. Nevertheless, multiple studies examining disease pathophysiology demonstrate that epigenetic dysregulation is a consistent feature of vitiligo, implicating DNA methylation, histone modifications, and ncRNAs as candidate regulatory layers that may stabilize melanocyte dysfunction and immune-mediated damage [123,124].

Mechanistic studies in patient-derived cells support this view. A recent study showed that CD8^+^ T cells from vitiligo patients exhibit epigenetically driven overexpression of perforin, which is associated with DNA hypomethylation of the *perforin 1* (*PRF1*) promoter locus and enhanced melanocyte cytotoxicity [125]. Complementary work demonstrated reproducible DNA methylation changes in vitiligo melanocytes and immune cells across cohorts and integrated these findings with transcriptome data from vitiligo melanocytes and lesional skin datasets. This revealed an overall negative correlation between methylation levels and differentially expressed genes. They proposed that changes in methylation level may regulate the differential expression of functional genes in vitiligo. To the best of our knowledge, this is the first study to analyze the methylation profile of vitiligo melanocytes. [126]. Additional studies highlight extensive dysregulation of circulating and cellular ncRNAs (including miRNAs and exosome-derived miRNAs) implicated in melanocyte function, oxidative stress responses, and immune regulation in vitiligo [127,128,129,130]. Oxidative stress and innate immune “training” appear to drive and stabilize many of these epigenetic changes, suggesting that effective therapy will likely require rational combination strategies (antioxidants plus epigenetic modulators plus targeted immune therapies) rather than single-agent approaches [123,131]. These findings highlight epigenetic candidate pathways; however, it remains to be determined whether they truly constitute therapeutic targets, as their epigenetic modulation has not yet been systematically evaluated and requires validation in additional experimental models and rigorously designed clinical studies.

### 3.4. Systemic Sclerosis (SSc)

SSc is a fibrotic autoimmune disease characterized by immune dysregulation, microvascular injury and progressive fibroblast-mediated extracellular matrix accumulation in skin and internal organs. The cutaneous manifestations of SSc include skin thickening, induration, and loss of elasticity, with skin fibrosis representing the hallmark and best-defined clinical feature of the disease [132]. While classical genetic risk factors account for only a limited proportion of disease susceptibility, increasing evidence demonstrates that epigenetic alterations in fibroblasts, endothelial cells, and immune cells mediate the transition from environmental or inflammatory triggers to persistent cutaneous fibrosis [50,133,134]. Pro-oxidant imbalance in SSc has also been documented and may be linked to systemic inflammation and endothelial injury [135]. In patients with SSc, aberrant DNA methylation patterns, histone modifications, and dysregulated ncRNA expression promote sustained activation of dermal fibroblasts, excessive extracellular matrix deposition, and impaired angiogenic responses [50,134]. These epigenetic changes directly contribute to fibroblast activation and collagen accumulation, linking immune and vascular abnormalities to progressive skin thickening and fibrosis. Key profibrotic pathways affected by epigenetic dysregulation include transforming growth factor beta (TGF-β) signaling, interferon-regulated gene networks, and pathways controlling vascular homeostasis, all of which are implicated in the development and maintenance of skin fibrosis in SSc [136]. Overall, SSc represents a paradigm in which stable epigenetic reprogramming integrates immune activation, vascular dysfunction, and fibroblast-driven skin fibrosis, providing a strong mechanistic framework for understanding cutaneous disease manifestations and identifying novel therapeutic targets.

Emerging studies have focused on specific HDAC isoforms. In an in vitro study, TSA markedly upregulated HDAC3 expression and reduced HDAC7 expression, leading to broad suppression of collagen gene expression [137]. However, selectively inhibiting HDAC7 with siRNA produced a more refined outcome; it markedly decreased type I and III collagen synthesis without triggering the upregulation of profibrotic mediators such as CTGF or ICAM-1 in SSc fibroblasts. These results suggest that HDAC7 knockdown offers a more targeted antifibrotic strategy than TSA, efficiently limiting extracellular matrix accumulation while avoiding the induction of additional fibrotic pathways. These data are purely preclinical, highlighting the need to develop a clinically available HDAC7-selective agent and explore how these insights can be translated into therapeutic strategies. At the level of histone methylation, the H3K27 methyltransferase EZH2 has emerged as a key epigenetic regulator of SSc pathology. Fibrotic progression was prevented by the EZH2 inhibitor 3-deazaneplanocin (DZNep) across both in vitro and in vivo settings [138]. In fibroblasts derived from SSc patients, escalating doses of DZNep led to a graded decrease in profibrotic transcripts and a marked reduction in cellular migration. In endothelial cells, DZNep restored normal angiogenic responses by activating the Notch pathway and upregulating delta-like ligand 4 (DLL4). In the bleomycin-induced skin fibrosis mouse model, EZH2 inhibition attenuated skin thickening, hydroxyproline content, and H3K27me3 presence in the skin.

An additional layer of histone-based regulation involves enhancer-associated epigenetic memory, whereby enhancers retain marks of prior activation, allowing chromatin to remain permissive and thereby sustaining gene expression over time. Chromatin profiling in patient-derived fibroblasts identified a TGF-β2 enhancer marked by active histone modifications (e.g., H3K27ac) that remains persistently active, maintaining high TGF-β2 expression and profibrotic gene programs even after inflammatory stimuli are withdrawn; this persistent enhancer activity depends on NF-κB and the bromodomain protein BRD4 [139]. The BRD4 inhibition through JQ1 suppressed TGF-β2 activity, reversed profibrotic gene expression, and reduced dermal fibrosis. In patient skin explants maintained in organ culture, JQ1 reduced TGF-β2 and COL1A1 signaling and decreased dermal collagen content. These findings suggest that BET/BRD4 inhibitors, and specifically JQ1, could erase pathological enhancer memory in SSc fibroblasts, although current BET inhibitors act systemically and are not specifically targeted to SSc and the affected tissues.

Overall, preclinical studies highlight histone-based epigenetic interventions as key strategies for modulating dermal fibrosis in SSc. Targeting of HDACs, EZH2, and BRDs governs profibrotic gene expression, fibroblast activation, and skin fibrosis in cellular and animal models. These therapeutic approaches underscore the relevance of targeting chromatin remodeling and enhancer memory to disrupt the persistence of fibrosis. However, within this context, there is currently no direct functional evidence supporting the efficacy of therapeutic interventions targeting DNA methylation changes or ncRNAs in SSc.

### 3.5. Lupus Erythematosus (LE)

LE is a chronic autoimmune disease with multi-organ involvement, including the skin, presenting as acute cutaneous LE (malar rash), subacute cutaneous LE (photosensitive annular or papulosquamous lesions), and chronic cutaneous LE, most commonly discoid lupus, which may result in scarring and alopecia [140]. Epigenetic mechanisms play a central role in linking environmental triggers to persistent immune-cell reprogramming and tissue-specific damage. Across systemic and cutaneous lupus, three major epigenetic patterns emerge: (i) DNA hypomethylation and altered DNMT activity in immune cells; (ii) dysregulation of histone-modifying enzymes, including HDACs and EZH2, which govern pathogenic T, B, and myeloid cell function; and (iii) ncRNA dysregulation that reinforces transcriptional programs and inflammatory pathways [141,142]. Multi-omic analyses of lupus skin reveal distinct programs in different cutaneous phenotypes, linking interferon-stimulated genes, keratinocyte stress responses, and hypomethylated regulatory regions, providing a rationale for lesion-specific therapeutic strategies [143,144,145]. However, studies directly evaluating epigenetic interventions in lupus skin remain limited.

Preclinical lupus models have provided clear proof of concept for HDAC inhibition. In lupus-prone mouse models (MRL/lpr and NZB/W F1), treatments with HDAC inhibitors reduced renal disease and shifted cytokine profiles toward reduced Th1/Th17 dominance and enhanced regulatory signaling [146]. Chromatin analyses demonstrated histone H3/H4 hypoacetylation in MRL/lpr splenocytes compared with controls and reported that HDAC inhibition can reset aberrant histone acetylation patterns in lupus models. Additionally, EZH2, the H3K27 methyltransferase, is overexpressed in systemic LE (SLE) T and B cells and contributes to pathogenic differentiation and cytokine production. Preclinical inhibition or knockdown of EZH2 reduced inflammatory cytokines and mortality in MRL/lpr mice [147]. At the same time, EZH2 inhibition can influence innate immune development. In addition to exerting broad effects on hematopoietic cells, EZH2 inhibitors have been reported to promote, including NK-cell expansion and enhanced cytotoxicity, highlighting both therapeutic potential and systemic impact. Ex vivo studies in SLE and subacute cutaneous lupus further demonstrate disease-associated DNA methylation abnormalities, including hypomethylation and overexpression of immune genes such as CD70, which contribute to autoreactive T-cell activation [141]. These studies suggest that HDAC-targeted strategies could serve as a foundation for exploring epigenetic interventions in dermal disease, including cutaneous lupus. Such approaches might benefit from selective and targeted delivery to minimize systemic toxicity and optimize local effects, supporting the concept that skin-focused applications could be built upon this preclinical evidence [143,144].

### 3.6. Lichen Planus (LP)

LP is a chronic inflammatory disorder affecting the skin and mucosal surfaces, characterized by basal keratinocyte apoptosis and epithelial hyperkeratosis [148]. Clinically, patients present with pruritic, violaceous papules in cutaneous LP (CLP) and erosive or reticular lesions in oral LP (OLP), with the latter carrying a risk of malignant transformation [149]. Although the immunopathology of LP has been extensively studied, particularly the role of cytotoxic T cells and epithelial stress responses, epigenetic mechanisms remain comparatively underexplored, especially in cutaneous disease. Available evidence suggests that epigenetic regulation may contribute to key pathological features of LP, including sustained inflammatory signaling, oxidative stress responses, keratinocyte apoptosis, and epithelial repair [149]. However, unlike psoriasis or AD, there are currently no studies that directly test epigenetic interventions in LP, and most data derive from observational or mechanistic studies in OLP. As such, LP represents an early-stage model in which epigenetics provides biological insight rather than established therapeutic strategies.

Early work on global DNA methylation in oral lichenoid disease suggested that large-scale DNA methylation alterations are not a defining feature of LP. In oral rinse samples using surrogates of genome-wide methylation, Bediaga and colleagues found no consistent shift towards either global hypomethylation or hypermethylation compared with normal mucosa [150]. In other words, the study did not identify a consistent global DNA methylation signature that distinguishes lichenoid disease from controls. These findings indicate that epigenetic changes in LP are more likely to be locus-specific, affecting selected inflammatory or differentiation genes. This implies that future DNMT-targeted therapies, if explored, will likely need to be highly focused rather than broad demethylating approaches. The study was cross-sectional and lacked gene-level methylation mapping or functional assays, so any therapeutic relevance remains limited.

Chromatin and DNA repair pathways have been examined more closely in comparative studies of OLP and CLP. In a retrospective cohort of 89 patients (66 OLP and 23 CLP), Gonzaga et al. quantified expression of base-excision repair proteins apurinic endonuclease 1 (APE1) and X-ray repair cross-complementing protein 1 (XRCC1), together with histone H3 lysine-9 acetylation (H3K9ac), using immunohistochemistry and immunofluorescence [151]. Immunoreactivity for APE1 and XRCC1 was significantly higher in CLP than in OLP, whereas H3K9ac showed a similar profile across oral versus cutaneous lesions, supporting H3K9 acetylation as a frequent epigenetic event in both conditions. Although the study did not test HDAC inhibitors or other chromatin-modifying agents, it suggests that H3K9ac and DNA repair proteins may be part of an adaptive epigenetic response.

Among epigenetic regulators, ncRNAs add an additional epigenetic layer, particularly in OLP, where miRNAs provide the most mechanistic evidence in LP. A detailed study by Ge and co-workers showed that miR-122 is markedly upregulated in OLP epithelium and in oral keratinocytes exposed to bacterial lipopolysaccharide (LPS) or activated CD4^+^ T-cell supernatants, via NF-κB–dependent signaling [152]. Functionally, miR-122 promoted keratinocyte apoptosis by directly targeting the vitamin D receptor (VDR), as confirmed by luciferase reporter assays. Loss of VDR expression amplified apoptotic signaling, while VDR overexpression mitigated both miR-122 induction and caspase-3 activation, establishing a miR-122–VDR regulatory axis linking inflammation to epithelial barrier breakdown. These findings identify miR-122 inhibition and VDR pathway restoration as conceptually attractive therapeutic strategies.

In summary, epigenetic dysregulation is clearly involved in LP pathophysiology, particularly through increased histone acetylation, activation of DNA repair responses, and pathogenic miRNA-mediated keratinocyte apoptosis. Nevertheless, no study currently provides direct evidence supporting epigenetic therapy in either CLP or OLP. Most available data focus on OLP, leaving CLP comparatively unexplored, despite its clinical relevance. Epigenetic alterations are present and biologically meaningful yet remain at the stage of target discovery rather than intervention. Future progress will require systematic epigenomic profiling of CLP lesions, development of appropriate experimental models, and functional testing of epigenetic modulators before targeted therapeutic strategies can be credibly pursued. Epigenetic interventions for the diseases described above are summarized in Table 1.

## 4. Knowledge Gaps and Future Directions: Toward Precision Epigenetic Therapies

Chronic inflammatory skin disorders, including psoriasis, AD, vitiligo, LE, SSc, and LP, arise from immune dysregulation and sustained inflammation [136,148,153,154]. Although mapping epigenetic alterations across these diseases has advanced, several critical knowledge gaps persist in translating these findings into therapeutic epigenetic strategies. Psoriasis and AD have been the primary focus of therapeutic epigenetic research, followed by vitiligo, whereas such treatment options for the cutaneous manifestations of other disorders are still at a very early stage of investigation.

Across existing studies, interventions targeting DNA methylation have been explored predominantly in psoriasis and vitiligo, mainly through the use of DNMT inhibitors such as 5-aza-dC derivatives, whereas their potential in other conditions remains poorly explored. Among histone-directed approaches, acetylation-based interventions have received comparatively greater attention. Selective inhibition of HDAC3 and HDAC6 has been investigated in AD, while several HDAC inhibitors, including vorinostat, entinostat, and remetinostat, have been studied in psoriasis. In SSc, therapeutic efforts have focused on selectively inhibiting HDAC7. In parallel, histone methylation-based therapies have been evaluated almost exclusively for the latter disease, primarily via inhibition of the methyltransferase EZH2, with GSK126 as an illustrative example. Although histone modification-based treatments have been described in lupus erythematosus, the available evidence largely pertains to systemic manifestations, with cutaneous involvement remaining insufficiently examined. In contrast, comparable approaches have not yet been explored in vitiligo or LP. BET protein inhibition has been assessed in only a limited number of conditions. Precisely, BET inhibitors have been studied in psoriasis, most notably JQ1, and in scleroderma, where research has focused particularly on BRD4, whereas such approaches remain unexplored in other diseases. Finally, therapeutic strategies targeting ncRNAs are scarce. MiRNA-based therapies have been examined mainly in psoriasis, with miR-125b as an illustrative example, while similar strategies are lacking in other conditions. To date, there are no reports of therapeutic targeting of lncRNAs in any of these skin diseases.

Viewed together, insights from individual disorders extend beyond their specific contexts, highlighting shared features that transcend disease boundaries. In many conditions, such as psoriasis, preclinical experimental models typically represent a single phenotype, leaving epigenetic interventions in other subtypes underexplored. Since many studies, particularly in LE, SSc, and LP, employ systemic or non-cutaneous models, additional skin-specific research is required to further support clinical translation. Moreover, bulk tissue analyses can obscure cell-specific epigenetic alterations, whereas single-cell approaches provide higher-resolution mechanistic insights [155]. In this context, studies should clarify the effects of epigenetic targeting on specific cell populations while minimizing toxicity in non-target cells [156]. Key questions remain regarding the cross-talk between epigenetic layers, their cell-type-specific functions, and their therapeutic potential [1]. Additionally, the potential impact of specific epigenetic interventions on skin homeostasis, regeneration, and immunosurveillance remains understudied. Addressing these gaps will require integrative approaches capable of capturing the multifactorial complexity of the disease, such as large-scale, longitudinal, multi-omics cohort studies. Building on these preclinical insights, it is essential to consider factors that may influence the translation of epigenetic therapies to widespread clinical practice. It remains poorly understood how factors such as age, prior inflammatory exposure, or long-term therapy influence the skin’s epigenetic responsiveness to interventions. Similarly, whether epigenetic therapies differ in efficacy between early and advanced disease stages also remains unclear, limiting optimization of treatment timing. Finally, whether patients’ epigenetic profiles can affect their response to currently available therapies warrants evaluation.

## 5. Conclusions

The present review delineates the current landscape of epigenetic therapeutic development across inflammatory and immune-mediated skin diseases, revealing pronounced differences in depth and translational maturity among conditions. Psoriasis has been studied most extensively, with multiple preclinical efforts targeting DNA methylation, histone acetylation, chromatin-associated regulatory proteins, and miRNA-based strategies. AD follows, with a growing body of work centered largely on histone deacetylase-directed interventions. By contrast, epigenetic therapeutic approaches in vitiligo remain largely indirect. SSc represents a distinct paradigm in which epigenetic modulation has demonstrated antifibrotic potential in preclinical models, although investigations focused specifically on cutaneous disease remain limited. Similarly, epigenetic interventions in LE and LP have been examined predominantly in systemic or mucosal settings, with skin-directed evidence remaining sparse. Collectively, these findings position epigenetic modulation as a biologically compelling and potentially versatile therapeutic avenue, while underscoring the need for broader, skin-focused interventional studies to enable durable clinical translation.

## Figures and Tables

**Table 1:** Epigenetic treatments in inflammatory and immune-mediated skin diseases.

Disease	Design	Experimental Model	Epigenetic Category	Class	Effect	Intervention	Outcome	Ref.
Psoriasis	In vivo	IMQ mouse model	DNA methylation	DNMTs	↓	5-aza-dC	Decreased DNMT1 expression and global DNA methylation; reversed DEHP-induced epigenetic and inflammatory alterations; downregulated IL-17A, IL-6 and iNOS; upregulated IL-10 and NRF2; reduced epidermal thickness	[47]
Psoriasis	In vivo	IMQ mouse model	Histone modifications	BRDs	↓	JQ1	Attenuated Th17 signature cytokines; suppressed RORC, IL-17A, and IL-22; reduced psoriasiform inflammation.	[90]
Psoriasis	In vitro; in vivo	HaCaT and U-937; IMQ mouse model	Histone modifications	BRDs	↓	OTX015; ABBV075	Reduced inflammatory gene expression; decreased IMQ-induced psoriasiform dermatitis in vivo.	[108]
Psoriasis	In vivo	IMQ mouse model	Histone modifications	BRDs	↓	9-fluorobenzo[f]pyrido [4,3-b][1,4]oxazepin-10-one derivative 43	Dose-dependent improvement in ear thickness; efficacy in the IMQ-induced psoriasis murine model.	[91]
Psoriasis	In vitro; in vivo	Primary human keratinocytes; psoriasis-like human skin xenografts on beige-SCID mice	Histone modifications	HDACs	↓	Vorinostat	Reduced keratinocyte hyperproliferation; promoted keratinocyte apoptosis and differentiation; alleviated psoriasiform pathology in the mouse model.	[109]
Psoriasis	In vitro; in vivo	Human CD4^+^ T cell, murine T cells, and NHEK; IMQ mouse model	Histone modifications	HDACs	↓	Entinostat	Attenuated psoriatic inflammation; Reduced IL-17A^+^ γδT cell infiltration; suppressed Th17 cell generation and psoriasis-related inflammatory mediator expression.	[110]
Psoriasis	In vitro in vivo	HaCaT and C57BL/6 male bone marrow cells; IMQ mouse model	Histone modifications	HDACs	↓	Remetinostat	Improved IMQ-induced psoriasiform dermatitis by suppressing dendritic cell maturation; reduced inflammatory cytokine expression; and normalized keratinocyte differentiation	[83]
Psoriasis	In vitro; in vivo	HaCaT IMQ mouse model	Non-coding RNAs	miRNAs	↑	FNA-miR-125b	Suppressed proliferation of HaCaT; decreased TNF-α, IL-6, IL-23, and STAT3 expression; reduced epidermal thickness and PASI score.	[112]
AD	In vivo	DNFB-induced NC/Nga mice	Histone modifications	HDACs	↓	TSA	Inhibited AD-like dermatitis; decreased IL-4 production; expansion of Treg cells.	[118]
AD	In vitro; in vivo	TNF-α/IFN-γ-treated HaCaT and Jurkat T cells; DNCB-induced BALB/c mice	Histone modifications	HDACs	↓	RGFP966	Upregulated NRF2/HO-1 signaling pathway; Reduced inflammation, immune dysfunction, and tissue damage.	[119]
AD	In vitro; in vivo	DNCB-treated HaCaT, NC/Nga murine dermal fibroblasts, and cutaneous mast cells; DNCB-induced NC/Nga mice; Oxazolone-induced SKH-1 mice	Histone modifications	HDACs	↓	TubA	Negative impact on the elevated release of histamine and serum prostaglandin E2 levels; Blocked epithelial hyperplasia and mast cell activation; Improved clinical symptoms.	[84]
SSc	In vitro	Normal and SSc human dermal fibroblasts	Histone modifications	HDACs	↓	TSA; siRNA	TSA mainly targeted HDAC3 and HDAC7 and suppressed collagen gene expression; selective silencing of HDAC7 by siRNA offers a more targeted antifibrotic strategy.	[137]
SSc	In vitro; in vivo	Human dermal fibroblasts and endothelial cells; bleomycin-induced C57BL/6 mice	Histone modifications	EZH2	↓	DZNep	Decreased profibrotic transcripts and cellular migration; restored normal angiogenic responses; attenuated skin thickening.	[138]
SSc	In vitro; ex vivo	Control-patient dermal fibroblasts and skin explants	Histone modifications	BRDs	↓	JQ1	Suppressed TGFB2 expression; reversed profibrotic gene expression programs.	[139]

5-aza-dC: 5-aza-2′-deoxycytidine, AD: Atopic dermatitis, BRDs: Bromodomains, DNCB: 2,4- dinitrochlorobenzene, DNFB: 2,4-dinitrofluorobenzene, DNMTs: DNA methyltransferases, DZNep: 3-deazaneplanocin, EZH2: Enhancer of zeste homolog 2, FNA: Functional nucleic acid, HaCaT: Human immortalized keratinocytes, HDACs: Histone deacetylases, iNOS: Inducible nitric oxide synthase, IL: Interleukin, IMQ: Imiquimod, miRNAs: MicroRNAs, NHEK: Normal human epidermal keratinocytes, NRF2: Nuclear factor erythroid 2-related factor 2, HO-1: Heme oxygenase 1, PASI: Psoriasis area and severity index, RORC: RAR-related orphan receptor C, SSc: Systemic sclerosis, siRNA: Small interfering RNA, TGFB2: Transforming growth factor beta 2, TSA: Trichostatin A, TubA: Tubastatin A. The downward arrow (↓) indicates downregulation of the corresponding activity, while the upward arrow (↑) indicates upregulation of the corresponding activity.

## Data Availability

No new data were created or analyzed in this study.

## Abbreviations

The following abbreviations are used in this manuscript:

5-Aza-dC	5-aza-2′-deoxycytidine
5hmC	5-hydroxymethylcytosine
5mC	5-methylcytosine
AD	Atopic dermatitis
APE1	Apurinic endonuclease 1
BET	Bromodomain and extra- terminal
BRD	Bromodomain
circRNA	Circular RNA
CLP	Cutaneous lichen planus
COL1A1	Collagen type I alpha 1 chain
CpG	Cytosine-guanine
CXCL13	C-X-C motif chemokine ligand 13
DEHP	Di-(2-ethylhexyl) phthalate
DLL4	Delta-like ligand 4
DNCB	2,4-dinitrochlorobenzene
DNFB	2,4-dinitrofluorobenzene
DNMT	DNA methyltransferase
DZNep	3-deazaneplanocin
EZH2	Enhancer of zeste homolog 2
FNA	Functional nucleic acid
H3K27me3	H3K27 trimethylation
H3K9ac	Histone H3 lysine-9 acetylation
HATs	Histone acetyltransferases
HDAC	Histone deacetylase
HO-1	Heme-oxygenase 1
IL	Interleukin
IMQ	Imiquimod
iNOS	Inducible nitric oxide synthase
LE	Lupus erythematosus
lncRNA	Long non-coding RNA
LP	Lichen planus
LPS	Lipopolysaccharide
miRNA	MicroRNA
ncRNA	Non-coding RNA
NF-κB	Nuclear factor kappa-light-chain enhancer of activated B cells
NK	Natural killer
NRF2	Nuclear factor erythroid 2-related factor 2
OLP	Oral lichen planus
PASI	Psoriasis area and severity index
PBMCs	Peripheral blood mononuclear cells
PRC	Polycomb repressive complex
PRF1	Perforin 1
PTMs	Post-translational modifications
RORC	RAR-related orphan receptor C
siRNA	Small interfering RNA
SIRT1	Sirtuin 1
SLE	Systemic lupus erythematosus
SSc	Systemic sclerosis
STAT3	Suppressing signal transducer and activator of transcription 3
TET	Ten-eleven translocation
TGF-β	Transforming growth factor beta
Th17	T helper 17 cells
TNF	Tumor necrosis factor
TSA	Trichostatin A
TubA	Tubastatin A
UTRs	Untranslated regions
VDR	Vitamin D receptor
XRCC1	X-ray repair cross-complementing protein 1

## References

[1] Lai X., Huang J., Li H., Chang C., Li R., Li X., Yan X., Dong L. (2025). Epigenetic Regulatory Mechanisms of Autoimmune Skin Diseases: Novel Biomarkers and Therapeutic Prospects. Clin. Epigenetics.

[2] Gibson F., Hanly A., Grbic N., Grunberg N., Wu M., Collard M., Alani R.M. (2022). Epigenetic Dysregulation in Autoimmune and Inflammatory Skin Diseases. Clin. Rev. Allergy Immunol..

[3] Möbus L., Weidinger S., Emmert H. (2020). Epigenetic Factors Involved in the Pathophysiology of Inflammatory Skin Diseases. J. Allergy Clin. Immunol..

[4] Huang M.Y., Armstrong A.W. (2023). Janus-Kinase Inhibitors in Dermatology: A Review of Their Use in Psoriasis, Vitiligo, Systemic Lupus Erythematosus, Hidradenitis Suppurativa, Dermatomyositis, Lichen Planus, Lichen Planopilaris, Sarcoidosis and Graft-versus-Host Disease. Indian J. Dermatol. Venereol. Leprol..

[5] Diotallevi F., Offidani A. (2023). Skin, Autoimmunity and Inflammation: A Comprehensive Exploration through Scientific Research. Int. J. Mol. Sci..

[6] Ujiie H., Rosmarin D., Schön M.P., Ständer S., Boch K., Metz M., Maurer M., Thaci D., Schmidt E., Cole C. (2022). Unmet Medical Needs in Chronic, Non-Communicable Inflammatory Skin Diseases. Front. Med..

[7] Schmidt A.D., De Guzman Strong C. (2021). Current Understanding of Epigenetics in Atopic Dermatitis. Exp. Dermatol..

[8] Ponikowska M., Vellone E., Czapla M., Uchmanowicz I. (2025). Challenges Psoriasis and Its Impact on Quality of Life: Challenges in Treatment and Management. Psoriasis Targets Ther..

[9] Leng M., Qi P., Li R., Gong F., Wei Z. (2025). Burden of Immune-Related Skin Diseases Worldwide, 1991–2021: Insights and Prediction from the Global Burden of Disease Study. Front. Immunol..

[10] Monteleone G., Moscardelli A., Colella A., Marafini I., Salvatori S. (2023). Immune-Mediated Inflammatory Diseases: Common and Different Pathogenic and Clinical Features. Autoimmun. Rev..

[11] Langan S.M., Irvine A.D., Weidinger S. (2020). Atopic Dermatitis. Lancet.

[12] Potestio L., Tommasino N., D’Agostino M., Esposito V., Lauletta G., Portarapillo A., Megna M. (2025). Biologics and Small Molecules for Psoriasis: Current and Future Progress. Drugs Context.

[13] Sutaria N., Au S.-C. (2021). Failure Rates and Survival Times of Systemic and Biologic Therapies in Treating Psoriasis: A Retrospective Study. J. Dermatol. Treat..

[14] Iskandar I.Y.K., Warren R.B., Lunt M., Mason K.J., Evans I., McElhone K., Smith C.H., Reynolds N.J., Ashcroft D.M., Griffiths C.E.M. (2018). Differential Drug Survival of Second-Line Biologic Therapies in Patients with Psoriasis: Observational Cohort Study from the British Association of Dermatologists Biologic Interventions Register (BADBIR). J. Investig. Dermatol..

[15] Burke O.M., Frerichs V.R., Garcia D.F., Stone R.C., Lev-Tov H., Czarnowicki T., Keane R.W., Ojeh N., Marjanovic J., Pastar I. (2025). The Impact of Innate Immunity and Epigenetics in the Pathogenesis of Hidradenitis Suppurativa. Front. Immunol..

[16] Da Silva Duarte A.J., Sanabani S.S. (2024). Deciphering Epigenetic Regulations in the Inflammatory Pathways of Atopic Dermatitis. Life Sci..

[17] Klibaner-Schiff E., Simonin E.M., Akdis C.A., Cheong A., Johnson M.M., Karagas M.R., Kirsh S., Kline O., Mazumdar M., Oken E. (2024). Environmental Exposures Influence Multigenerational Epigenetic Transmission. Clin. Epigenetics.

[18] Mateu-Arrom L., Puig L. (2023). Genetic and Epigenetic Mechanisms of Psoriasis. Genes.

[19] Tiffon C. (2018). The Impact of Nutrition and Environmental Epigenetics on Human Health and Disease. Int. J. Mol. Sci..

[20] Sabounchi S., Bollyky J., Nadeau K. (2015). Review of Environmental Impact on the Epigenetic Regulation of Atopic Diseases. Curr. Allergy Asthma Rep..

[21] Dilshat R., Vu H.N., Steingrímsson E. (2021). Epigenetic Regulation during Melanocyte Development and Homeostasis. Exp. Dermatol..

[22] Leśniak W. (2021). Epigenetic Regulation of Epidermal Differentiation. Epigenomes.

[23] Stylianides C., Hadjigavriel G., Theotokis P., Vakirlis E., Meditskou S., Manthou M.E., Dermitzakis I. (2025). Epigenetic Mechanisms in Neurofibromatosis Types 1 and 2. Epigenomes.

[24] Hadjigavriel G., Stylianides C., Axarloglou E., Manthou M.E., Vakirlis E., Theotokis P., Meditskou S., Dermitzakis I. (2025). Epigenetic Insights into Tuberous Sclerosis Complex, Von Hippel-Lindau Syndrome, and Ataxia-Telangiectasia. Epigenomes.

[25] Nardacchione E.M., Tricarico P.M., Moura R., d’Adamo A.P., Thasneem A., Suleman M., Marzano A.V., Crovella S., Moltrasio C. (2023). Unraveling the Epigenetic Tapestry: Decoding the Impact of Epigenetic Modifications in Hidradenitis Suppurativa Pathogenesis. Genes.

[26] Ghosh A., Himaja A., Biswas S., Kulkarni O., Ghosh B. (2023). Advances in the Delivery and Development of Epigenetic Therapeutics for the Treatment of Cancer. Mol. Pharm..

[27] Yuan M., Lee J., Taylor M., Cho R.J., Cheng J.B. (2025). Advancing Precision Medicine in Inflammatory Skin Disease. Am. J. Clin. Dermatol..

[28] Daccache J.A., Naik S. (2024). Inflammatory Memory in Chronic Skin Disease. JID Innov..

[29] Dermitzakis I., Chatzi D., Kyriakoudi S.A., Evangelidis N., Vakirlis E., Meditskou S., Theotokis P., Manthou M.E. (2024). Skin Development and Disease: A Molecular Perspective. Curr. Issues Mol. Biol..

[30] Kasprowicz-Furmańczyk M., Narbutt J., Borzęcki A., Owczarczyk-Saczonek A. (2023). Does Molecular Scarring in Psoriasis Exist? A Review of the Literature. Postep. Dermatol. Alergol..

[31] Sawada Y., Gallo R.L. (2021). Role of Epigenetics in the Regulation of Immune Functions of the Skin. J. Investig. Dermatol..

[32] Tsou P.-S., Varga J., O’Reilly S. (2021). Advances in Epigenetics in Systemic Sclerosis: Molecular Mechanisms and Therapeutic Potential. Nat. Rev. Rheumatol..

[33] Moltrasio C., Romagnuolo M., Marzano A.V. (2022). Epigenetic Mechanisms of Epidermal Differentiation. Int. J. Mol. Sci..

[34] Reolid A., Muñoz-Aceituno E., Abad-Santos F., Ovejero-Benito M.C., Daudén E. (2021). Epigenetics in Non-Tumor Immune-Mediated Skin Diseases. Mol. Diagn. Ther..

[35] Venkatesh S., Workman J.L. (2015). Histone Exchange, Chromatin Structure and the Regulation of Transcription. Nat. Rev. Mol. Cell Biol..

[36] Fang C., Yu P., Li J., Chen X., Peng C. (2025). Epigenetics of Atopic Dermatitis: Pathogenesis and Therapeutic Prospects. Curr. Opin. Immunol..

[37] Moore L.D., Le T., Fan G. (2013). DNA Methylation and Its Basic Function. Neuropsychopharmacology.

[38] Angeloni A., Bogdanovic O. (2021). Sequence Determinants, Function, and Evolution of CpG Islands. Biochem. Soc. Trans..

[39] Deaton A.M., Bird A. (2011). CpG Islands and the Regulation of Transcription. Genes Dev..

[40] Jeltsch A., Jurkowska R.Z. (2014). New Concepts in DNA Methylation. Trends Biochem. Sci..

[41] Del Castillo Falconi V.M., Torres-Arciga K., Matus-Ortega G., Díaz-Chávez J., Herrera L.A. (2022). DNA Methyltransferases: From Evolution to Clinical Applications. Int. J. Mol. Sci..

[42] Tajima S., Suetake I., Takeshita K., Nakagawa A., Kimura H., Song J., Jeltsch A., Jurkowska R.Z. (2022). Domain Structure of the Dnmt1, Dnmt3a, and Dnmt3b DNA Methyltransferases. DNA Methyltransferases—Role and Function.

[43] Lyko F. (2018). The DNA Methyltransferase Family: A Versatile Toolkit for Epigenetic Regulation. Nat. Rev. Genet..

[44] Edwards J.R., Yarychkivska O., Boulard M., Bestor T.H. (2017). DNA Methylation and DNA Methyltransferases. Epigenetics Chromatin.

[45] Ito S., D’Alessio A.C., Taranova O.V., Hong K., Zhang Y. (2012). Role of Tet Proteins in 5mC to 5hmC Conversion, ES Cell Self-Renewal, and ICM Specification. Nature.

[46] Wu H., Zhang Y. (2011). Mechanisms and Functions of Tet Protein-Mediated 5-Methylcytosine Oxidation. Genes Dev..

[47] Alfardan A.S., Nadeem A., Ahmad S.F., Al-Harbi N.O., Alqinyah M., Attia S.M., El-Sherbeeny A.M., Al-Harbi M.M., Al-Shabanah O.A., Ibrahim K.E. (2024). DNMT Inhibitor, 5-Aza-2′-Deoxycytidine Mitigates Di(2-Ethylhexyl) Phthalate-Induced Aggravation of Psoriasiform Inflammation in Mice via Reduction in Global DNA Methylation in Dermal and Peripheral Compartments. Int. Immunopharmacol..

[48] Christman J.K. (2002). 5-Azacytidine and 5-Aza-2′-Deoxycytidine as Inhibitors of DNA Methylation: Mechanistic Studies and Their Implications for Cancer Therapy. Oncogene.

[49] Jackson-Grusby L., Laird P.W., Magge S.N., Moeller B.J., Jaenisch R. (1997). Mutagenicity of 5-Aza-2′-Deoxycytidine Is Mediated by the Mammalian DNA Methyltransferase. Proc. Natl. Acad. Sci. USA.

[50] Tsou P.-S. (2019). Epigenetic Control of Scleroderma: Current Knowledge and Future Perspectives. Curr. Rheumatol. Rep..

[51] Bannister A.J., Kouzarides T. (2011). Regulation of Chromatin by Histone Modifications. Cell Res..

[52] Kouzarides T. (2007). Chromatin Modifications and Their Function. Cell.

[53] Jenuwein T., Allis C.D. (2001). Translating the Histone Code. Science.

[54] Strahl B.D., Allis C.D. (2000). The Language of Covalent Histone Modifications. Nature.

[55] Yi J.Z., McGee J.S. (2021). Epigenetic-modifying Therapies: An Emerging Avenue for the Treatment of Inflammatory Skin Diseases. Exp. Dermatol..

[56] Kablan T., Biyikli E., Bozdemir N., Uysal F. (2025). A Narrative Review of the Histone Acetylation and Deacetylation during Mammalian Spermatogenesis. Biochimie.

[57] Milazzo G., Mercatelli D., Di Muzio G., Triboli L., De Rosa P., Perini G., Giorgi F.M. (2020). Histone Deacetylases (HDACs): Evolution, Specificity, Role in Transcriptional Complexes, and Pharmacological Actionability. Genes.

[58] Gallinari P., Marco S.D., Jones P., Pallaoro M., Steinkühler C. (2007). HDACs, Histone Deacetylation and Gene Transcription: From Molecular Biology to Cancer Therapeutics. Cell Res..

[59] Ghosh K., O’Neil K., Capell B.C. (2018). Histone Modifiers: Dynamic Regulators of the Cutaneous Transcriptome. J. Dermatol. Sci..

[60] Verdone L., Caserta M., Mauro E.D. (2005). Role of Histone Acetylation in the Control of Gene Expression. Biochem. Cell Biol..

[61] Seto E., Yoshida M. (2014). Erasers of Histone Acetylation: The Histone Deacetylase Enzymes. Cold Spring Harb. Perspect. Biol..

[62] Annunziato A.T., Hansen J.C. (2001). Role of Histone Acetylation in the Assembly and Modulation of Chromatin Structures. Gene Expr..

[63] Filippakopoulos P., Knapp S. (2014). Targeting Bromodomains: Epigenetic Readers of Lysine Acetylation. Nat. Rev. Drug Discov..

[64] Jambhekar A., Dhall A., Shi Y. (2019). Roles and Regulation of Histone Methylation in Animal Development. Nat. Rev. Mol. Cell Biol..

[65] Michalak E.M., Burr M.L., Bannister A.J., Dawson M.A. (2019). The Roles of DNA, RNA and Histone Methylation in Ageing and Cancer. Nat. Rev. Mol. Cell Biol..

[66] Di Croce L., Helin K. (2013). Transcriptional Regulation by Polycomb Group Proteins. Nat. Struct. Mol. Biol..

[67] Margueron R., Reinberg D. (2011). The Polycomb Complex PRC2 and Its Mark in Life. Nature.

[68] Zhang J., Jing L., Li M., He L., Guo Z. (2019). Regulation of Histone Arginine Methylation/Demethylation by Methylase and Demethylase (Review). Mol. Med. Rep..

[69] Kim J.-H., Lee J., Lee I.-S., Lee S., Cho K. (2017). Histone Lysine Methylation and Neurodevelopmental Disorders. Int. J. Mol. Sci..

[70] Teng Y., Zou M., Zhou X., Wu J., Liu S., Yuan Z., Jia Y., Zhang K., Li X., Ye J. (2022). Novel Prospects for Scarless Wound Healing: The Roles of Myofibroblasts and Adipocytes. J. Cell. Mol. Med..

[71] Moritz L.E., Trievel R.C. (2018). Structure, Mechanism, and Regulation of Polycomb-Repressive Complex 2. J. Biol. Chem..

[72] Schuettengruber B., Bourbon H.-M., Di Croce L., Cavalli G. (2017). Genome Regulation by Polycomb and Trithorax: 70 Years and Counting. Cell.

[73] Cedar H., Bergman Y. (2009). Linking DNA Methylation and Histone Modification: Patterns and Paradigms. Nat. Rev. Genet..

[74] Viré E., Brenner C., Deplus R., Blanchon L., Fraga M., Didelot C., Morey L., Van Eynde A., Bernard D., Vanderwinden J.-M. (2006). The Polycomb Group Protein EZH2 Directly Controls DNA Methylation. Nature.

[75] Greer E.L., Shi Y. (2012). Histone Methylation: A Dynamic Mark in Health, Disease and Inheritance. Nat. Rev. Genet..

[76] Black J.C., Van Rechem C., Whetstine J.R. (2012). Histone Lysine Methylation Dynamics: Establishment, Regulation, and Biological Impact. Mol. Cell.

[77] Zeng J., Zhang J., Sun Y., Wang J., Ren C., Banerjee S., Ouyang L., Wang Y. (2022). Targeting EZH2 for Cancer Therapy: From Current Progress to Novel Strategies. Eur. J. Med. Chem..

[78] Zhou C., Zhao D., Wu C., Wu Z., Zhang W., Chen S., Zhao X., Wu S. (2024). Role of Histone Deacetylase Inhibitors in Non-Neoplastic Diseases. Heliyon.

[79] Shi J., Vakoc C.R. (2014). The Mechanisms behind the Therapeutic Activity of BET Bromodomain Inhibition. Mol. Cell.

[80] Glaser K.B. (2007). HDAC Inhibitors: Clinical Update and Mechanism-Based Potential. Biochem. Pharmacol..

[81] Hassell K.N. (2019). Histone Deacetylases and Their Inhibitors in Cancer Epigenetics. Diseases.

[82] Leus N.G.J., Van Der Wouden P.E., Van Den Bosch T., Hooghiemstra W.T.R., Ourailidou M.E., Kistemaker L.E.M., Bischoff R., Gosens R., Haisma H.J., Dekker F.J. (2016). HDAC 3-Selective Inhibitor RGFP966 Demonstrates Anti-Inflammatory Properties in RAW 264.7 Macrophages and Mouse Precision-Cut Lung Slices by Attenuating NF-κB P65 Transcriptional Activity. Biochem. Pharmacol..

[83] Jin L., Jiang Q., Huang H., Zhou X. (2024). Topical Histone Deacetylase Inhibitor Remetinostat Improves IMQ-Induced Psoriatic Dermatitis via Suppressing Dendritic Cell Maturation and Keratinocyte Differentiation and Inflammation. Eur. J. Pharmacol..

[84] Kwon Y., Choi Y., Kim M., Jeong M.S., Jung H.S., Jeoung D. (2021). HDAC6 and CXCL13 Mediate Atopic Dermatitis by Regulating Cellular Interactions and Expression Levels of miR-9 and SIRT1. Front. Pharmacol..

[85] Ghiboub M., Elfiky A.M.I., De Winther M.P.J., Harker N.R., Tough D.F., De Jonge W.J. (2021). Selective Targeting of Epigenetic Readers and Histone Deacetylases in Autoimmune and Inflammatory Diseases: Recent Advances and Future Perspectives. J. Pers. Med..

[86] Eckschlager T., Plch J., Stiborova M., Hrabeta J. (2017). Histone Deacetylase Inhibitors as Anticancer Drugs. Int. J. Mol. Sci..

[87] Falkenberg K.J., Johnstone R.W. (2014). Histone Deacetylases and Their Inhibitors in Cancer, Neurological Diseases and Immune Disorders. Nat. Rev. Drug Discov..

[88] Kim H.-J., Bae S.-C. (2011). Histone Deacetylase Inhibitors: Molecular Mechanisms of Action and Clinical Trials as Anti-Cancer Drugs. Am. J. Transl. Res..

[89] Ali H.A., Li Y., Bilal A.H.M., Qin T., Yuan Z., Zhao W. (2022). A Comprehensive Review of BET Protein Biochemistry, Physiology, and Pathological Roles. Front. Pharmacol..

[90] Nadeem A., Al-Harbi N.O., Al-Harbi M.M., El-Sherbeeny A.M., Ahmad S.F., Siddiqui N., Ansari M.A., Zoheir K.M.A., Attia S.M., Al-Hosaini K.A. (2015). Imiquimod-Induced Psoriasis-like Skin Inflammation Is Suppressed by BET Bromodomain Inhibitor in Mice through RORC/IL-17A Pathway Modulation. Pharmacol. Res..

[91] Sato M., Kondo T., Kohno Y., Seto S. (2021). Discovery of Benzo[f]Pyrido[4,3-b][1,4]Oxazepin-10-One Derivatives as Orally Available Bromodomain and Extra-Terminal Domain (BET) Inhibitors with Efficacy in an In Vivo Psoriatic Animal Model. Bioorg. Med. Chem..

[92] Dermitzakis I., Kyriakoudi S.A., Chatzianagnosti S., Chatzi D., Vakirlis E., Meditskou S., Manthou M.E., Theotokis P. (2025). Epigenetics in Skin Homeostasis and Ageing. Epigenomes.

[93] Surace A.E.A., Hedrich C.M. (2019). The Role of Epigenetics in Autoimmune/Inflammatory Disease. Front. Immunol..

[94] Bure I.V., Nemtsova M.V., Kuznetsova E.B. (2022). Histone Modifications and Non-Coding RNAs: Mutual Epigenetic Regulation and Role in Pathogenesis. Int. J. Mol. Sci..

[95] Peschansky V.J., Wahlestedt C. (2014). Non-Coding RNAs as Direct and Indirect Modulators of Epigenetic Regulation. Epigenetics.

[96] Bartel D.P. (2009). MicroRNAs: Target Recognition and Regulatory Functions. Cell.

[97] Morales S., Monzo M., Navarro A. (2017). Epigenetic Regulation Mechanisms of microRNA Expression. Biomol. Concepts.

[98] Yao Q., Chen Y., Zhou X. (2019). The Roles of microRNAs in Epigenetic Regulation. Curr. Opin. Chem. Biol..

[99] Statello L., Guo C.-J., Chen L.-L., Huarte M. (2021). Gene Regulation by Long Non-Coding RNAs and Its Biological Functions. Nat. Rev. Mol. Cell Biol..

[100] Poltronieri P. (2024). Regulatory RNAs: Role as Scaffolds Assembling Protein Complexes and Their Epigenetic Deregulation. Explor. Target. Anti-Tumor Ther..

[101] Yang Z., Xu F., Teschendorff A.E., Zhao Y., Yao L., Li J., He Y. (2022). Insights into the Role of Long Non-Coding RNAs in DNA Methylation Mediated Transcriptional Regulation. Front. Mol. Biosci..

[102] Armstrong A.W., Blauvelt A., Callis Duffin K., Huang Y.-H., Savage L.J., Guo L., Merola J.F. (2025). Psoriasis. Nat. Rev. Dis. Primers.

[103] Wang L., Liu R., Tang Y., Ma Y., Wang G., Ruan Q., Zheng S.J., Wang L., Liu R., Tang Y. (2025). Advances in Psoriasis Research: Decoding Immune Circuits and Developing Novel Therapies. Int. J. Mol. Sci..

[104] Gudjonsson J.E., Krueger G. (2012). A Role for Epigenetics in Psoriasis: Methylated Cytosine–Guanine Sites Differentiate Lesional from Nonlesional Skin and from Normal Skin. J. Investig. Dermatol..

[105] Pollock R.A., Abji F., Gladman D.D. (2017). Epigenetics of Psoriatic Disease: A Systematic Review and Critical Appraisal. J. Autoimmun..

[106] Guo J., Zheng Q., Peng Y. (2023). BET Proteins: Biological Functions and Therapeutic Interventions. Pharmacol. Ther..

[107] Hall J.A., Pokrovskii M., Kroehling L., Kim B.-R., Kim S.Y., Wu L., Lee J.-Y., Littman D.R. (2022). Transcription Factor RORα Enforces Stability of the Th17 Cell Effector Program by Binding to a Rorc Cis-Regulatory Element. Immunity.

[108] Wu X., Shi Z., Hsu D.K., Chong J., Huynh M., Mendoza L., Yamada D., Hwang S.T. (2020). A Monocyte-Keratinocyte-Derived Co-Culture Assay Accurately Identifies Efficacies of BET Inhibitors as Therapeutic Candidates for Psoriasiform Dermatitis. J. Dermatol. Sci..

[109] Samuelov L., Bochner R., Magal L., Malovitski K., Sagiv N., Nousbeck J., Keren A., Fuchs-Telem D., Sarig O., Gilhar A. (2022). Vorinostat, a Histone Deacetylase Inhibitor, as a Potential Novel Treatment for Psoriasis. Exp. Dermatol..

[110] Jiang Y., Lu S., Lai Y., Wang L. (2023). Topical Histone Deacetylase 1 Inhibitor Entinostat Ameliorates Psoriasiform Dermatitis through Suppression of IL-17A Response. J. Dermatol. Sci..

[111] Ovejero-Benito M.C., Reolid A., Sánchez-Jiménez P., Saiz-Rodríguez M., Muñoz-Aceituno E., Llamas-Velasco M., Martín-Vilchez S., Cabaleiro T., Román M., Ochoa D. (2018). Histone Modifications Associated with Biological Drug Response in Moderate-to-Severe Psoriasis. Exp. Dermatol..

[112] Han Y., Xi L., Leng F., Xu C., Zheng Y. (2024). Topical Delivery of microRNA-125b by Framework Nucleic Acids for Psoriasis Treatment. Int. J. Nanomed..

[113] Frazier W., Bhardwaj N. (2020). Atopic Dermatitis: Diagnosis and Treatment. Am. Fam. Physician.

[114] Li H., Zhang Z., Zhang H., Guo Y., Yao Z. (2021). Update on the Pathogenesis and Therapy of Atopic Dermatitis. Clin. Rev. Allergy Immunol..

[115] Martin M.J., Estravís M., García-Sánchez A., Dávila I., Isidoro-García M., Sanz C., Martin M.J., Estravís M., García-Sánchez A., Dávila I. (2020). Genetics and Epigenetics of Atopic Dermatitis: An Updated Systematic Review. Genes.

[116] Chen C., Zeng J., Lu J. (2023). Critical Role of Epigenetic Modification in the Pathogenesis of Atopic Dermatitis. Indian J. Dermatol. Venereol. Leprol..

[117] Shibata S. (2023). Epigenetic Control of Skin Immunity. Immunol. Med..

[118] Kim T.-H., Jung J.-A., Kim G.-D., Jang A.-H., Cho J.-J., Park Y.S., Park C.-S. (2010). The Histone Deacetylase Inhibitor, Trichostatin A, Inhibits the Development of 2,4-Dinitrofluorobenzene-Induced Dermatitis in NC/Nga Mice. Int. Immunopharmacol..

[119] Zhou W., Zeng D., Liu S., Huang Y., Lv F., Zhou W. (2024). Histone Deacetylase 3 Inhibition Alleviates 2,4-Dinitrochlorobenzene-Induced Atopic Dermatitis via Epigenetically Upregulating Nrf2/HO-1 Signaling Pathway. Int. Immunopharmacol..

[120] Speeckaert R., Van Caelenberg E., Belpaire A., Speeckaert M.M., van Geel N. (2024). Vitiligo: From Pathogenesis to Treatment. J. Clin. Med..

[121] Bergqvist C., Ezzedine K. (2021). Vitiligo: A Focus on Pathogenesis and Its Therapeutic Implications. J. Dermatol..

[122] Seneschal J., Bae J.M., Ezzedine K., Hamzavi I., Harris J.E., Bellei B., Parsad D., Passeron T., van Geel N., Boniface K. (2025). Vitiligo. Nat. Rev. Dis. Primers.

[123] He H., Wang T. (2025). Vitiligo and Epigenetics: From Pathogenesis to Clinical Applications. Exp. Dermatol..

[124] Wu L., Han T., Wang Y., Li S., Li C. (2025). Epigenetic Regulation in Vitiligo: Mechanisms, Challenges, and Therapeutic Opportunities. Curr. Opin. Immunol..

[125] Deng Q., Zou P., Du P., Shi Y., Pi Z., Xiao Y., Kanekura T., Zhang H., Zhan Y., Qiu X. (2023). Overexpressed Perforin Contributes to the Melanocyte Destruction via Epigenetic Regulation in Patients with Vitiligo. Int. Immunopharmacol..

[126] Pu Y., Chen X., Chen Y., Zhang L., Chen J., Zhang Y., Shao X., Chen J. (2021). Transcriptome and Differential Methylation Integration Analysis Identified Important Differential Methylation Annotation Genes and Functional Epigenetic Modules Related to Vitiligo. Front. Immunol..

[127] Abdallah H.Y., Faisal S., Tawfik N.Z., Soliman N.H., Kishk R.M., Ellawindy A. (2023). Expression Signature of Immune-Related MicroRNAs in Autoimmune Skin Disease: Psoriasis and Vitiligo Insights. Mol. Diagn. Ther..

[128] AbdElneam A.I., Mohammed G.F. (2025). Non-Coding RNAs (miRNAs–circRNAs–lncRNAs) and Genes Interact with the Regulation of Vitiligo. Arch. Dermatol. Res..

[129] Li L. (2020). The Role of MicroRNAs in Vitiligo: Regulators and Therapeutic Targets. Ann. Dermatol..

[130] Li W., Pang Y., He Q., Song Z., Xie X., Zeng J., Guo J. (2024). Exosome-Derived microRNAs: Emerging Players in Vitiligo. Front. Immunol..

[131] Tian J., Wang Y., Ding M., Zhang Y., Chi J., Wang T., Jiao B., Jian Z., Yi X., Huang Y. (2021). The Formation of Melanocyte Apoptotic Bodies in Vitiligo and the Relocation of Vitiligo Autoantigens under Oxidative Stress. Oxid. Med. Cell. Longev..

[132] Herrick A.L., Assassi S., Denton C.P. (2022). Skin Involvement in Early Diffuse Cutaneous Systemic Sclerosis: An Unmet Clinical Need. Nat. Rev. Rheumatol..

[133] Ramahi A., Altorok N. (2020). Epigenetics and Systemic Sclerosis: An answer to disease onset and evolution?. Eur. J. Rheumatol. Rep..

[134] Yu J., Tang R., Ding K. (2022). Epigenetic Modifications in the Pathogenesis of Systemic Sclerosis. Int. J. Gen. Gen. Med..

[135] Dziedzic R., Wójcik K., Olchawa M., Sarna T., Pięta J., Jakieła B., Padjas A., Korona A., Zaręba L., Potaczek D.P. (2023). Increased Oxidative Stress Response in Circulating Blood of Systemic Sclerosis Patients—Relation to Disease Characteristics and Inflammatory Blood Biomarkers. Semin. Arthritis Rheum..

[136] Son H.-H., Moon S.-J. (2024). Pathogenesis of Systemic Sclerosis: An Integrative Review of Recent Advances. J. Rheum. Dis..

[137] Hemmatazad H., Rodrigues H.M., Maurer B., Brentano F., Pileckyte M., Distler J.H.W., Gay R.E., Michel B.A., Gay S., Huber L.C. (2009). Histone Deacetylase 7, a Potential Target for the Antifibrotic Treatment of Systemic Sclerosis. Arthritis Rheum..

[138] Tsou P.-S., Campbell P., Amin M.A., Coit P., Miller S., Fox D.A., Khanna D., Sawalha A.H. (2019). Inhibition of EZH2 Prevents Fibrosis and Restores Normal Angiogenesis in Scleroderma. Proc. Natl. Acad. Sci. USA.

[139] Shin J.Y., Beckett J.D., Bagirzadeh R., Creamer T.J., Shah A.A., McMahan Z., Paik J.J., Sampedro M.M., MacFarlane E.G., Beer M.A. (2019). Epigenetic Activation and Memory at a TGFB2 Enhancer in Systemic Sclerosis. Sci. Transl. Med..

[140] Ameer M.A., Chaudhry H., Mushtaq J., Khan O.S., Babar M., Hashim T., Zeb S., Tariq M.A., Patlolla S.R., Ali J. (2022). An Overview of Systemic Lupus Erythematosus (SLE) Pathogenesis, Classification, and Management. Cureus.

[141] Jeffries M.A., Sawalha A.H. (2011). Epigenetics in Systemic Lupus Erythematosus: Leading the Way for Specific Therapeutic Agents. Int. J. Clin. Rheumtol..

[142] Xiao G., Zuo X. (2016). Epigenetics in Systemic Lupus Erythematosus. Biomed. Rep..

[143] Adams D.E., Shao W.-H., Adams D.E., Shao W.-H. (2022). Epigenetic Alterations in Immune Cells of Systemic Lupus Erythematosus and Therapeutic Implications. Cells.

[144] Araki Y., Mimura T. (2024). Epigenetic Dysregulation in the Pathogenesis of Systemic Lupus Erythematosus. Int. J. Mol. Sci..

[145] Li Q., Jia C., Pan W., Liu H., Tang C., Weber D., Chen K., Long H., Byrne-Steele M.L., Han J. (2024). Multi-Omics Study Reveals Different Pathogenesis of the Generation of Skin Lesions in SLE and IDLE Patients. J. Autoimmun..

[146] Reilly C.M., Regna N., Mishra N. (2011). HDAC Inhibition in Lupus Models. Mol. Med..

[147] Yang Y., Liu K., Liu M., Zhang H., Guo M. (2022). EZH2: Its Regulation and Roles in Immune Disturbance of SLE. Front. Pharmacol..

[148] Vičić M., Hlača N., Kastelan M., Brajac I., Sotošek V., Massari L. (2023). Comprehensive Insight into Lichen Planus Immunopathogenesis. Int. J. Mol. Sci..

[149] Tekin B., Xie F., Lehman J.S. (2024). Lichen Planus: What Is New in Diagnosis and Treatment?. Am. J. Clin. Dermatol..

[150] Bediaga N., Marichalar-Mendia X., Aguirre-Urizar J., Calvo B., Echebarria-Goicouria M., de Pancorbo M., Acha-Sagredo A. (2014). Global DNA Methylation: Uncommon Event in Oral Lichenoid Disease. Oral Dis..

[151] Gonzaga A.K.G., Lopes M.L.D.d.S., Squarize C.H., Castilho R.M., de Medeiros A.M.C., Rocha K.B.F., da Silveira É.J.D. (2020). Expression Profile of DNA Repair Proteins and Histone H3 Lys-9 Acetylation in Cutaneous and Oral Lichen Planus. Arch. Oral Biol..

[152] Ge X., Xie H., Wang L., Li R., Zhang F., Xu J., Zhao B., Du J. (2021). MicroRNA-122 Promotes Apoptosis of Keratinocytes in Oral Lichen Planus through Suppressing VDR Expression. J. Cell. Mol. Med..

[153] Campione E., Lanna C., Diluvio L., Cannizzaro M.V., Grelli S., Galluzzo M., Talamonti M., Annicchiarico-Petruzzelli M., Mancini M., Melino G. (2020). Skin Immunity and Its Dysregulation in Atopic Dermatitis, Hidradenitis Suppurativa and Vitiligo. Cell Cycle.

[154] Danilenko D.M. (2016). An Overview of the Pathogenesis of Immune-Mediated Skin Injury. Toxicol. Pathol..

[155] Kim J., Detmar M. (2024). Stromal Cells and Epigenetics: Emerging Key Players of Chronic Inflammatory Skin Diseases. BMB Rep..

[156] Pan J., Chen S., Chen X., Song Y., Cheng H. (2025). Histone Modifications and DNA Methylation in Psoriasis: A Cellular Perspective. Clin. Rev. Allergy Immunol..

